# Damsel: analysis and visualisation of DamID sequencing in R

**DOI:** 10.1093/bioinformatics/btae695

**Published:** 2024-11-18

**Authors:** Caitlin G Page, Andrew Lonsdale, Katrina A Mitchell, Jan Schröder, Kieran F Harvey, Alicia Oshlack

**Affiliations:** Peter MacCallum Cancer Centre, Melbourne, 3000, Australia; Sir Peter MacCallum Department of Oncology, The University of Melbourne, Parkville, 3010, Australia; Peter MacCallum Cancer Centre, Melbourne, 3000, Australia; Sir Peter MacCallum Department of Oncology, The University of Melbourne, Parkville, 3010, Australia; Murdoch Children’s Research Institute, Parkville, 3052, Australia; Peter MacCallum Cancer Centre, Melbourne, 3000, Australia; Sir Peter MacCallum Department of Oncology, The University of Melbourne, Parkville, 3010, Australia; Computational Sciences Initiative, Department of Microbiology and Immunology, Peter Doherty Institute for Infection and Immunity, The University of Melbourne, Melbourne, 3000, Australia; Peter MacCallum Cancer Centre, Melbourne, 3000, Australia; Sir Peter MacCallum Department of Oncology, The University of Melbourne, Parkville, 3010, Australia; Department of Anatomy and Developmental Biology, Biomedicine Discovery Institute, Monash University, Clayton, 3800, Australia; Peter MacCallum Cancer Centre, Melbourne, 3000, Australia; Sir Peter MacCallum Department of Oncology, The University of Melbourne, Parkville, 3010, Australia; School of Mathematics and Statistics, The University of Melbourne, Parkville, 3010, Australia

## Abstract

**Summary:**

DamID sequencing is a technique to map the genome-wide interaction of a protein with DNA. Damsel is the first Bioconductor package to provide an end to end analysis for DamID sequencing data within R. Damsel performs quantification and testing of significant binding sites along with exploratory and visual analysis. Damsel produces results consistent with previous analysis approaches.

**Availability and implementation:**

The R package Damsel is available for install through the Bioconductor project https://bioconductor.org/packages/release/bioc/html/Damsel.html and the code is available on GitHub https://github.com/Oshlack/Damsel/.

## 1 Introduction

Defining the binding landscape of chromatin interacting proteins is vital for understanding and manipulating gene expression. DamID sequencing (DamID-seq) was developed as an alternative to ChIP-seq that facilitates this without relying on antibodies (van Steensel and Henikoff 2000). In the DamID-seq protocol, the *Escherichia coli* DNA adenine methylase (Dam) protein is fused to a transcription regulatory protein of interest, and upon interaction with DNA, Dam methylates the adenine in adjacent GATC motifs. As Dam can also randomly methylate GATC motifs, DamID-seq analysis is always conducted with the expression of a Dam-only control. Dam-methylated genomic regions are enriched using a methylation sensitive endonuclease to cut DNA and amplified with PCR. The ends of the resultant DNA fragments are sequenced and mapped back to regions between known GATC motifs within the genome to provide the genome-wide binding profile of the protein of interest. Following the identification of enriched genomic regions relative to the Dam-only control, potential target genes of the protein of interest can be identified. These target genes are identified based on their proximity to an enriched binding peak. A limitation of DamID-seq is the reliance on GATC motifs for defining the potential enriched regions, which limits the utility of DamID-seq to genetically tractable species, and makes it ill suited for identifying histone modifications, as well as modifications in GATC motif sparse regions in the genome. Targeted DamID-seq is an optimized version of DamID-seq that was developed in *Drosophila melanogaster* which allows in vivo expression and precise spatiotemporal control (Southall *et al.* 2013, Marshall *et al.* 2016, Aughey *et al.* 2019).

Although conceptually DamID-seq is similar to ChIP-seq, the region based nature of DNA fragmentation means that the data produced is different, requiring the development of new analytical tools. Bespoke analyses are commonly conducted, as in Vissers *et al.* (2018) and the R package Daim (Ashmore 2019) (available on GitHub) which utilize edgeR’s (Robinson *et al.* 2010) robust statistical testing. A 2019 review of DamID-seq mentions only two published methods (Li *et al.* 2015, Marshall and Brand 2015), both focussed on algorithms to facilitate peak calling (identifying regions of binding compared with the control) (Aughey *et al.* 2019). Neither of these tools conduct the whole DamID-seq bioinformatics workflow, thereby forcing researchers to rely on combining multiple tools and platforms with bespoke analysis. The widely used method, damidseq_pipeline (Marshall and Brand 2015), is the most comprehensive tool and runs on the command line analyzing samples individually, but has no visualization capabilities. Here, we present Damsel, the first dedicated R Bioconductor package for DamID sequencing, providing an end-to-end analysis, including exploratory and visualization capabilities.

## 2 Methods

**Figure 1.**
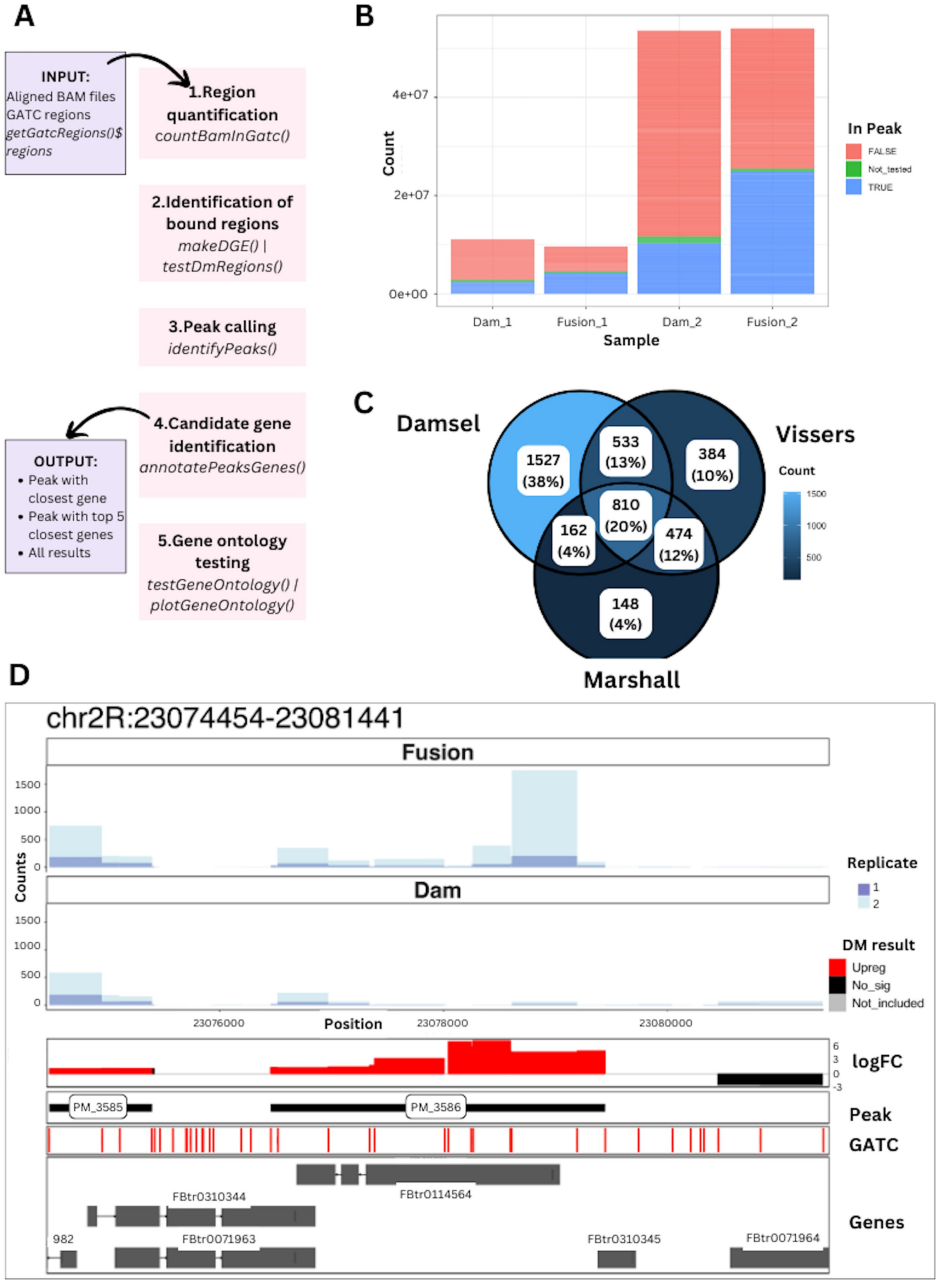
Overview of Damsel. (A) The main steps in the Damsel workflow alongside their Bioconductor functions, including region quantification, identification of bound regions, peak calling, candidate gene detection, and gene ontology testing. (B) Quality control plot illustrating the total counts (sequencing depth) of the samples, coloured by if the count from the region was part of the statistical testing, and if it was included in a peak. The replicate 1 samples (Dam_1 & Fusion_1) have a much lower sequencing library than replicate 2. (C) Venn diagram of peak overlap from the analysis of Vissers *et al.* (2018) data by Damsel, Vissers pipeline, and damidseq_pipeline (Marshall and Brand 2015). As expected, Damsel has a greater overlap with Vissers as they rely on the same testing procedure (edgeR) than it does with damidseq_pipeline. (D) Example visualization available in Damsel of the most significant peak found within the Vissers data. The layers of the plot show the counts across the regions for the Fusion and Dam-only samples, the differential methylation test results, the presence of significant peaks, the positions of the GATC sites, and the overlap to gene annotations.

The Damsel workflow, as described in Fig. 1A, starts after read alignment using standard tools (Supplementary Methods) and requires BAM files and GATC regions for a genome as input. Damsel conducts the following steps in the analysis workflow: region quantification, identification of bound genomic regions, peak calling, candidate gene detection, and gene ontology testing, described in more detail below.

### 2.1 Region quantification

The genome is intrinsically partitioned into genomic regions demarcated by GATC motifs (note that GATC is its own reverse complement and therefore the motif demarcates both strands of the DNA simultaneously). Damsel then utilizes the *featureCount()* function from the Rsubread package (Liao *et al.* 2019) to summarize the read counts between two adjacent GATC sites (GATC region) from the provided BAM files and motif input file. For paired-end BAM files, Damsel instead summarizes the fragments (read pairs). Due to the background methylation that occurs in the Dam-only sample, there is high correlation of the read counts of the regions between samples (Supplementary Fig. S1).

### 2.2 Identification of bound genomic regions

Previously, enrichment of a bound genomic region was identified from the log2 ratio of the region counts of the fusion sample compared to the control. However, analysis by Marshall and Brand (2015) established that this can result in a negative bias, highlighting the importance of normalization. Damsel first filters out excessively large regions (>10 000 bp), along with low count regions, before normalization and statistical testing is conducted with edgeR (McCarthy *et al.* 2012, Chen *et al.* 2016, Chen *et al.* 2024) allowing statistical testing incorporating the replicates and with arbitrary experimental designs (Robinson *et al.* 2010). edgeR’s TMM normalization assumes the majority of regions are not significant (Robinson and Oshlack 2010) and differential testing utilizes a quasi-likelihood negative binomial model (Robinson *et al.* 2010), utilising experimental replicates to model the biological variance in the data.

### 2.3 Peak calling

Peak calling allows for aggregation of adjacent significant regions that are likely to be the result of the same binding event. Damsel identifies peaks by aggregating the region level differential expression results, with *P*-value threshold set to 0.01 (by default), and log2 fold change threshold set to one. If the gap to the next significant region is less than 150 bp, it is included in the peak. Regions smaller than this size have consistently lower counts, and thus represent a greater proportion of regions that are excluded from differential testing. Peaks are ranked based on the statistics for ChIP-seq analysis presented in csaw (Lun and Smyth 2014, Lun and Smyth 2016) using the region with the lowest *P*-value to represent the peak and ordering peak significance based on this *P*-value. It should be noted that this approach has been tuned on data arising from transcription factor Dam-fusions rather than larger chromatin binding regions which might require further investigations.

### 2.4 Candidate gene identification

Methods for associating peaks and genes match the peak to its closest gene, with distance taken from the middle of the peak to the transcription start site. This is straightforward if the analysis is conducted on a species with little overlap between genes, like humans. However in species like *Drosophila melanogaster*, there is a large amount of overlap between genes and the closest gene may not be the most likely candidate. Therefore, Damsel outputs each peak with a list of candidate target genes, along with their genomic positions and relevant statistics. Placing the genes within the context of the peaks provides a more informative output, and sets Damsel apart from other tools.

At this point, Damsel outputs a file with all identified peaks and their associated statistics, matched to their associated candidate genes. Comparatively, damidseq_pipeline (Marshall and Brand 2015) outputs a list of genes and a score, which is the end of the analysis. However, Damsel also includes the following utilities.

### 2.5 Gene ontology testing

Gene ontology testing can be used to interpret the set of candidate target genes identified from the peaks. Damsel repurposes goseq, an R package developed to correct for detection biases in RNA-seq (Young *et al.* 2010) This is important because the GATC motifs are not spread evenly throughout the genome and different genes have different lengths and therefore a different number of GATC regions associated with them. This results in a bias where genes with more GATC regions are more likely to be associated with a peak (binding event) (Supplementary Fig. S2). Without bias correction, the gene ontology terms identified are not truly representative of the data. goseq takes as input a list of genes, if they are significant, and the number of GATC regions within 2 kb of the gene as the bias parameter, and outputs a list of overrepresented gene ontology terms.

### 2.6 Visualizations

A unique feature of Damsel is its visualization capability. Other DamID sequencing analysis methods, including damidseq_pipeline (Marshall and Brand 2015), output files that they recommend viewing in IGV, requiring researchers to switch software platforms. Damsel provides the opportunity to use its output files to create plots within R. Building on ggbio (Yin *et al.* 2012) and ggcoverage (Song and Wang 2023), Damsel contains ggplot2 (Wickham 2016) style plots that present results from different stages of the analysis. These plots can be layered, allowing the visualization of counts per region, log2 fold change and differential expression testing *P*-values, peak location, GATC motif, and gene positions for a provided region (Fig. 1D).

## 3 Results

To evaluate Damsel, we applied it to a subset of the data presented in Vissers *et al.* (2018). This dataset contained two Dam only samples (Dam_1, Dam_2) and two samples with Dam fused to *Scalloped* (Fusion_1, Fusion_2) in Drosophila wing tissue (Vissers *et al.* 2018). Initial QC plots from Damsel revealed differences in the sequencing depth between replicates (Fig. 1B). Damsel’s results were compared to the Marshall and Brand (2015) pipeline, and the Vissers *et al.* (2018) bespoke pipeline. Daim (Ashmore 2019) was excluded as it was not able to run. Due to not being actively maintained, the code for the Vissers’ analysis and peak calling had to be updated manually, and Marshall and Brand’s results had to be manually compiled to obtain one peak set. It should also be noted that the output of Vissers’ peak script did not contain individual *P*-values or FDRs for each reported peak, limiting some downstream analyses.

While all three approaches conduct the main steps of DamID-Seq analysis (identifying regions of enrichment, combining regions into peaks, and associating peaks with genes), Damsel provides increased functionality and seamless data flow through the pipeline (Supplementary Table S1). Like Vissers *et al.* (2018) analysis, Damsel incorporates replicates into the analysis to model variability and significantly reduce the burden of compiling results. However, Damsel can further incorporate complex designs within the edgeR framework, including pairing of replicates. Additional features unique to Damsel include region quantification directly from bam files, the ranking of significant results (peaks and genes), providing relevant statistics associating the peaks with annotated genes, and capacity for visualization of the results (Supplementary Table S1).

To first evaluate the false positive rate, the samples from Vissers *et al.* (2018) experiment were reassigned labels as Fusion and Dam-only so that instead of comparing the Dam-only to the fusion we compared one fusion and one Dam-only sample to the other fusion and Dam-only sample. There are two possible configurations of this. In the Dam_1 + Fusion_1 versus Dam_2 + Fusion_2 case, Damsel detected no significant peaks but Vissers falsely identified 1254 significant regions and 186 peaks (FDR < 0.01). When switching the samples to compare Dam_1 + Fusion_2 versus Dam_2 + Fusion_1 case, neither Damsel or Vissers identified significant regions or peaks, demonstrating good Type 1 error control (Supplementary Methods).

We next analysed the data in the way it was intended with two replicates of the fusion versus Dam_only. In Damsel, we included a factor for the replicate in the design. Damsel was found to be more sensitive than the original reported results finding 3120 peaks, which is 834 more than identified from running Vissers *et al.* (2018) pipeline. Damsel successfully identified 60% of Vissers’ and Marshall and Brand’s peaks (Fig. 1C). Both Marshall and Vissers’ pipelines do not include a factor for the replicates during the testing, which may explain why their results have a higher proportion of overlap. However, we argue that Damsel’s incorporation of the replicates is necessary to account for the clear difference in the coverage between the replicates (Fig. 1B). As not all experiments will have this relationship between the replicates, this is an optional element of Damsel’s testing procedure. Damsel’s gene ontology (GO) testing accounts for the bias in the number of regions per gene (Supplementary Fig. S2). We performed GO analysis on the peaks reported in the original paper and those found by Damsel and reassuringly found that Damsel reported 6 of the same top 10 GO terms as the regions from Vissers (Supplementary Fig. S3). Finally, as an example of data visualization we used Damsel to show the data and analysis of the most significant peak identified by all three methods (Fig. 1D).

Identifying which method is the most sensitive and accurate is beyond the scope of this study, and would be difficult without a large amount of experimental validation. However, Damsel performs consistently compared with established methods, and has produced results on multiple published datasets.

## 4 Conclusions

Damsel offers an end-to-end DamID-seq analysis pipeline including exploratory analysis and visualizations. On published data we find similar results to previous bespoke analysis. We find that Damsel can control false discovery rate and is more sensitive. Damsel includes new features such as the ranking of peak significance and performing gene ontology analysis. Future work will include the utility to perform motif enrichment analysis on sequences found in enriched peaks.

## Supplementary Material

btae695_Supplementary_Data

## Data Availability

The data used in this paper is available at the European Nucleotide Archive (PRJNA494322) https://www.ebi.ac.uk/ena/browser/view/PRJNA494322. Analysis presented in this paper can be found at https://caitlinpage.github.io/damsel_paper/.
