# Correction to: Damsel: analysis and visualisation of DamID sequencing in R

**DOI:** 10.1093/bioinformatics/btae753

**Published:** 2024-12-28

This is a correction to: Caitlin G Page, Andrew Lonsdale, Katrina A Mitchell, Jan Schröder, Kieran F Harvey, Alicia Oshlack, Damsel: analysis and visualisation of DamID sequencing in R, *Bioinformatics*, Volume 40, Issue 12, December 2024, btae695, https://doi.org/10.1093/bioinformatics/btae695

In the originally published version, the second author was incorrectly listed as Andrew Londsdale. This has been corrected to read Andrew Lonsdale.

